# Association between smoking status and outcomes in myocardial infarction patients undergoing percutaneous coronary intervention

**DOI:** 10.1038/s41598-021-86003-w

**Published:** 2021-03-19

**Authors:** Ching-Hui Sia, Junsuk Ko, Huili Zheng, Andrew Fu-Wah Ho, David Foo, Ling-Li Foo, Patrick Zhan-Yun Lim, Boon Wah Liew, Ping Chai, Tiong-Cheng Yeo, Huay-Cheem Tan, Terrance Chua, Mark Yan-Yee Chan, Jack Wei Chieh Tan, Heerajnarain Bulluck, Derek J. Hausenloy

**Affiliations:** 1 Department of Cardiology, National University Heart Centre Singapore, Singapore, Singapore; 2 Yong Loo Lin School of Medicine, National University of Singapore, Singapore, Singapore; 3 MD Program, Duke-NUS Medical School, Singapore, Singapore; 4 Health Promotion Board, National Registry of Diseases Office, Singapore, Singapore; 5 SingHealth Duke-NUS Emergency Medicine Academic Clinical Programme, Singapore, Singapore; 6 National Heart Research Institute Singapore, National Heart Centre Singapore, Singapore, Singapore; 7 Pre-Hospital and Emergency Care Research Centre, Health Services and Systems Research, Duke-NUS Medical School, Singapore, Singapore; 8 Department of Cardiology, National Heart Centre Singapore, Singapore, Singapore; 9 Tan Tock Seng Hospital, Singapore, Singapore; 10 Khoo Teck Puat Hospital, Singapore, Singapore; 11 Changi General Hospital, Singapore, Singapore; 12 Norfolk and Norwich University Hospital, Norwich, UK; 13 Norwich Medical School, University of East Anglia, Norwich, UK; 14 Cardiovascular and Metabolic Disorders Program, Duke-National University of Singapore Medical School, 8 College Road, Level 8, Singapore, 169857 Singapore; 15 The Hatter Cardiovascular Institute, University College London, London, UK; 16 Cardiovascular Research Center, College of Medical and Health Sciences, Asia University, Taichung City, Taiwan

**Keywords:** Cardiology, Cardiovascular diseases

## Abstract

Smoking is one of the leading risk factors for cardiovascular diseases, including ischemic heart disease and hypertension. However, in acute myocardial infarction (AMI) patients, smoking has been associated with better clinical outcomes, a phenomenon termed the “smoker’s paradox.” Given the known detrimental effects of smoking on the cardiovascular system, it has been proposed that the beneficial effect of smoking on outcomes is due to age differences between smokers and non-smokers and is therefore a smoker’s pseudoparadox. The aim of this study was to evaluate the association between smoking status and clinical outcomes in ST-segment elevation (STEMI) and non-STEMI (NSTEMI) patients treated by percutaneous coronary intervention (PCI), using a national multi-ethnic Asian registry. In unadjusted analyses, current smokers had better clinical outcomes following STEMI and NSTEMI. However, after adjusting for age, the protective effect of smoking was lost, confirming a smoker’s pseudoparadox. Interestingly, although current smokers had increased risk for recurrent MI within 1 year after PCI in both STEMI and NSTEMI patients, there was no increase in mortality. In summary, we confirm the existence of a smoker’s pseudoparadox in a multi-ethnic Asian cohort of STEMI and NSTEMI patients and report increased risk of recurrent MI, but not mortality, in smokers.

## Introduction

Cardiovascular disease (CVD) is the leading cause of death worldwide accounting for about 30% of deaths^1^. CVD is an imminent health threat as the number of global deaths from CVD has been rapidly increasing due to the aging population^2–4^. Among the various CVDs, acute myocardial infarction (AMI) results in a significant 30-day mortality of between 3 and 14%^5^.

Smoking is one of the strongest risk factors for cardiovascular disease including AMI^6^. However, paradoxically, a number of clinical studies have documented that smokers might have a better prognosis following AMI events as compared to non-smokers in both patients with ST-segment elevation (STEMI) and non-ST-segment elevation myocardial infarction (NSTEMI)^7,8^. To explain this seemingly counter-intuitive beneficial effect of smoking on AMI, a number of hypotheses have been proposed; (1) preconditioning of cardiomyocytes, (2) cellular reprograming from necrosis to apoptosis^9^, and (3) reduced impact of platelets^10^. Of these, the preconditioning phenomenon has been most supported due to the established role of preconditioning on the regeneration of cardiomyocytes^11^. The size of the infarct is strongly associated with patient outcomes, such as all-cause mortality and hospitalization after STEMI^12^. Based on this observation and the possibility that cigarette smoking may mimic a transient preconditioning stimulus, it has been proposed that smoking leads to a better outcome via preconditioning in cardiomyocytes and, therefore, decreases the size of infarction^13,14^.

The results of previously published observational studies demonstrating beneficial effects of smoking and post-AMI outcomes have been challenged^7,8^. In both of these reports, the average age of smokers was about 9 years younger than that of non-smokers. Despite the fact that aging is one of the risk factors of AMI, the age difference was not adjusted in the studies. Therefore, it is still possible that the observed “protection” was presumably due to younger age of the smokers rather than smoking itself^15^. The role of cigarette smoking on the outcomes of STEMI and NSTEMI patient remains controversial and has implications on public health. As such, to further clarify this issue, a national population-based multi-ethnic Asian acute myocardial infarction registry was used to evaluate the associations between smoking status and clinical outcomes in STEMI and NSTEMI patients treated by percutaneous coronary intervention.

## Methods

### Data collection

For this study, data from a national registry, the Singapore Myocardial Infarction Registry (SMIR), were utilized. The institutional review board granted an exemption for conducting this study without need for informed consent (SingHealth Centralised Institutional Review Board Reference No: 2016/2480) as this study involved analysis of a dataset without identifiers. The research was conducted in accordance with the Declaration of Helsinki. The SingHealth Centralised Institutional Review Board approved the research, and all research was performed in accordance with relevant guidelines/regulations. The SMIR collects epidemiology and clinical data of AMI cases diagnosed in the public and private hospitals in Singapore, in addition to certified out-of-hospital AMI deaths^16–18^. Reporting of AMI cases to this registry is mandated by law in accordance with the National Registry of Diseases Act. The registry data included patient medical claim listings, hospital in-patient discharge summaries and cardiac biomarker listings from hospital laboratories. To identify the cases of AMI, the International Classification of Diseases, Ninth Revision, Clinical Modification (ICD-9-CM) code 410 was used for the data collected between 2007 and 2011. For any data that were collected from 2012 onwards, ICD-10 (Australian Modification) codes I21 and I22 were used to identify AMI. The AMI cases were further classified into STEMI and NSTEMI based on diagnosis documented by the clinicians in the medical records. The following criteria were used to define STEMI: (1) chest pain for 20 min, (2) significant ST-segment elevation, and (3) positive for cardiac biomarkers. To ensure that the data were captured in an accurate and consistent manner across all hospitals over the years, annual internal audit was performed. Among the STEMI and NSTEMI cases in January 2007 to December 2015, information on the patients who received PCI was extracted and utilized for analysis. Only patients who received PCI were included as the patients with a Type 1 MI were the focus of this study.

### Clinical outcomes

The primary clinical outcome of this study was all-cause mortality at 1-year post-presentation. Mortality data were obtained from the Death Registry of Ministry of Home Affairs and were merged with the SMIR data. Secondary outcomes included all-cause mortality at 30 days post-presentation, and the first episode of MI occurring within 1 year after the index PCI. The analyses were stratified by the type of AMI (STEMI and NSTEMI) and patients were compared based on their smoking status (never smoked, former smoker, and current smoker). Smoking data were self-reported by the patients or their family based on documentation in the medical records.

### Statistical analysis

Categorical variables were expressed as frequency and percentages, while continuous variables were expressed as median and interquartile range. Missing data were excluded from the analyses through case deletion without imputation to maintain data in its original form. Univariable and multivariable cox regression were performed to determine the hazards ratios (HR) of having the primary and secondary outcomes. Specifically for the secondary outcome of recurrent AMI, competing risk from non-AMI deaths was adjusted using the Fine-Gray proportional hazards model^19^.

This study did not require informed consent according to the exemption granted by the institutional review board as this was a study using deidentified data (SingHealth CIRB Reference No: 2016/2480). This study followed the principles of the Declaration of Helsinki. The statistician of the study had access to anonymized individual data, while the other co-authors had access to the analyzed aggregated data. All analyses were done using Stata SE Version 13 (StataCorp. 2013. Stata Statistical Software: Release 13. College Station, TX: StataCorp LP). All statistical tests were 2-tailed and results were deemed to be statistically significant if p < 0.05.

## Results

### Study population

**Figure 1:**
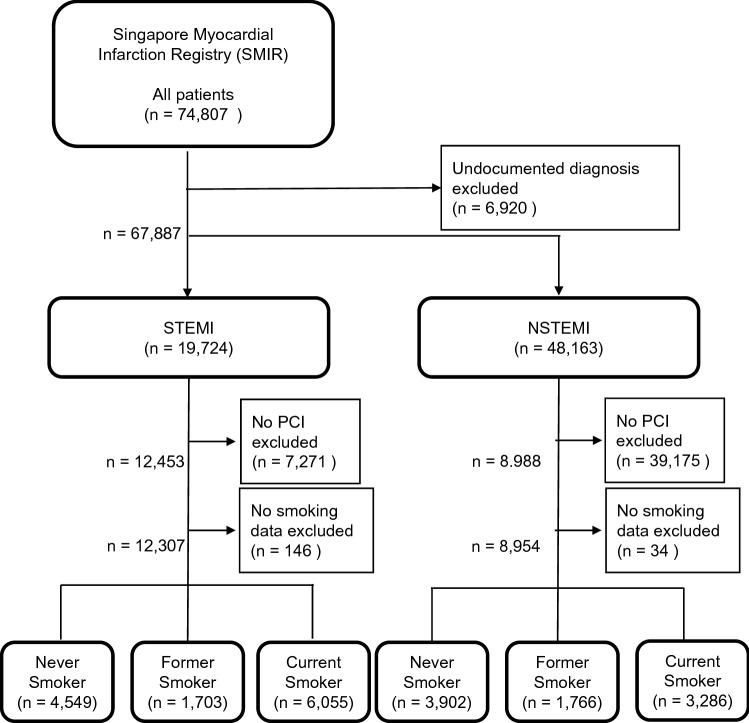
Flowchart of data inclusion. 74,807 patients were registered in the Singapore Myocardial Infarction Registry (SMIR). 6920 patients were excluded from the data analysis as the type of their acute coronary syndrome (ACS) was unclear. This exclusion left only ST elevation myocardial infarction (STEMI) and Non-STEMI (NSTEMI) in the dataset. Those who did not undergo percutaneous coronary intervention (PCI) or did not have smoking data were excluded from the data analysis. The patients were then further divided into the details of their smoking status.

A total of 21,261 AMI patients (12,307 STEMI and 8,954 NSTEMI) who received PCI from January 2007 to December 2015 were included in this study (Fig. 1). The SMIR population was multi-ethnic with about three-fifths being Chinese, one-fifth Malays and one-fifth Indian (Tables 1 and 2). The STEMI group comprised 4549 (37%) never smoked, 1703 (14%) former smokers, and 6055 (49%) current smokers (Table 1). The median age of patients who never smoked or were former smokers were similar, but the median age of current smokers was significantly less by 8 years. The NSTEMI group comprised 3902 (44%) never smoked, 1766 (20%) former smokers, and 3286 (37%) current smokers (Table 2). The median age of current smokers with NSTEMI patients was about 9 years less than the never smoked and former smokers. For both STEMI and NSTEMI, former and current smokers were predominantly male (> 90%), when compared to patients who have never smoked (50–60%) (Tables 1 and 2). The female patients with STEMI had a worse 1-year-mortality (HR 1.33, 95% CI 1.05–1.69), suggesting that gender was a potential confounding factor. Therefore, gender was included for adjustment when the multivariate regression was performed.

**Table 1:** Demographic and clinical characteristics of STEMI patients by smoking status.

	Never smoker (n = 4549)	Former smoker (n = 1703)	Current smoker (n = 6055)	p-value
Age in years, median (IQR)	61.3 (53.8–70.8)	61.1 (53.2–70.3)	54.4 (48.0–61.1)	< 0.001
Male, n (%)	2966 (65.2)	1633 (95.9)	5876 (97.0)	< 0.001
**Ethnicity, n (%)**				
Chinese	3057 (67.2)	1057 (62.0)	3500 (57.8)	< 0.001
Malay	647 (14.2)	371 (21.8)	1453 (24.0)	
Indian	778 (17.1)	235 (13.8)	997 (16.5)	
Others	67 (1.5)	40 (2.4)	105 (1.7)	
History of diabetes, n (%)	1573 (34.6)	567 (33.3)	1316 (21.7)	< 0.001
History of hypertension, n (%)	2867 (63.0)	1042 (61.3)	2491 (41.2)	< 0.001
History of hyperlipidemia, n (%)	2341 (51.5)	948 (55.7)	2331 (38.5)	< 0.001
History of AMI/CABG/PCI, n (%)	587 (12.9)	410 (24.1)	846 (14.0)	< 0.001
BMI in kg/m^2^, median (IQR)	24.5 (22.3–27.2)	24.9 (22.6–27.4)	24.5 (22.3–27.3)	0.035
**Killip class on admission, n (%)**				
I	3712 (81.5)	1426 (83.8)	5109 (84.4)	< 0.001
II	217 (4.8)	89 (5.2)	289 (4.8)	
III	203 (4.5)	70 (4.1)	182 (3.0)	
IV	416 (9.2)	117 (6.9)	473 (7.8)	
CPR in ambulance/ED, n (%)	198 (4.4)	49 (2.9)	199 (3.3)	0.003
Serum creatinine in 10 µmol on admission, median (IQR)	9.0 (7.4–11.2)	9.6 (8.2–11.6)	8.9 (7.7–10.4)	< 0.001
Haemoglobin in g/dL on admission, median (IQR)	14.0 (12.7–15.2)	14.5 (13.3–15.4)	15.1 (14.1–16.1)	< 0.001
Left ventricular ejection fraction < 50%, n (%)	2629 (62.4)	1030 (64.4)	3384 (59.9)	0.001
Anterior infarct, n (%)	2300 (50.6)	814 (47.8)	3048 (50.3)	0.127
Symptom-to-balloon time in minutes, median (IQR)	193 (125–330)	196 (122–322)	180 (120–301)	< 0.001

*AMI acute myocardial infarction, *BMI* body mass index, *CABG* coronary artery bypass surgery, *CPR* cardiopulmonary resuscitation, *ED* emergency department, *IQR* interquartile range, *PCI* percutaneous coronary intervention, *STEMI* ST-elevation myocardial infarction.

**Table 2:** Demographic and clinical characteristics of NSTEMI patients by smoking status.

	Never smoker (n = 3902)	Former smoker (n = 1766)	Current smoker (n = 3286)	p-value
Age in years, median (IQR)	63.5 (55.7–72.6)	63.6 (55.9–72.5)	54.9 (48.8–62.2)	< 0.001
Male, n (%)	2134 (54.7)	1658 (93.9)	3150 (95.9)	< 0.001
**Ethnicity, n (%)**				
Chinese	2567 (65.7)	1107 (62.7)	1896 (57.7)	< 0.001
Malay	568 (14.6)	339 (19.2)	733 (22.3)	
Indian	695 (17.8)	293 (16.6)	603 (18.4)	
Others	72 (1.9)	27 (1.5)	54 (1.6)	
History of diabetes, n (%)	1828 (46.9)	771 (43.7)	895 (27.3)	< 0.001
History of hypertension, n (%)	2997 (76.8)	1344 (76.1)	1724 (52.5)	< 0.001
History of hyperlipidemia, n (%)	2676 (68.6)	1294 (73.4)	1802 (54.8)	< 0.001
History of AMI/CABG/PCI, n (%)	1163 (29.8)	836 (47.3)	877 (26.7)	< 0.001
BMI in kg/m^2^, median (IQR)	24.9 (22.5–28.0)	24.8 (22.4–27.5)	25.1 (22.8–27.9)	0.031
**Killip class on admission, n (%)**				
I	3171 (81.2)	1380 (78.2)	2924 (89.0)	< 0.001
II	431 (11.1)	221 (12.5)	210 (6.4)	
III	256 (6.6)	142 (8.1)	112 (3.4)	
IV	43 (1.1)	22 (1.3)	40 (1.2)	
CPR in ambulance/ED, n (%)	25 (0.6)	10 (0.6)	15 (0.5)	0.579
Serum creatinine in 10 µmol on admission, median (IQR)	8.6 (7.1–11.3)	9.5 (7.9–12.4)	8.4 (7.2–9.7)	< 0.001
Haemoglobin in g/dL on admission, median (IQR)	13.3 (11.8–14.6)	13.9 (12.4–15.0)	14.8 (13.8–15.7)	< 0.001
Left ventricular ejection fraction < 50%, n (%)	1226 (39.0)	671 (47.5)	970 (36.2)	< 0.001

*AMI acute myocardial infarction, *BMI* body mass index, *CABG* coronary artery bypass surgery, *CPR* cardiopulmonary resuscitation, *ED* emergency department, *IQR* interquartile range, *NSTEMI* non ST-elevation myocardial infarction, *PCI* percutaneous coronary intervention.

### Clinical outcomes

In the STEMI group, the unadjusted HRs for death within 1 year were significantly lower for current smokers (HR 0.50), when compared to never smokers (Table 3). For death within 30 days, the unadjusted HRs were significantly lower in both former (HR 0.68) and current (HR 0.50) smokers, when compared to never smokers (Table 3). The Kaplan Meier analysis also displayed the beneficial effect of smoking on the three primary outcomes in STEMI and NSTEMI patients (Supplementary Figure S1). However, after adjustment for potential confounders, this protective effect was no longer observed (Table 3). Similar 30-day and 1-year mortality results were observed when we stratified the analysis by ethnicity (Table 3). Notably, although the unadjusted HRs for recurrent MI within 1 year did not differ significantly according to smoking status, the adjusted HR for current smokers (HR 1.39) was significantly higher than never smokers, especially among Chinese patients, suggesting that current smokers have increased risk of recurrent MI following STEMI.

**Table 3:** Risk of death within 30 days, death within 1 year and recurrent MI within 1 year among former and current smokers compared to never smokers in STEMI patients.

	Death within 30 days		Death within 1 year		Recurrent MI within 1 year	
	Unadjusted HR (95% CI)	Adjusted HR* (95% CI)	Unadjusted HR (95% CI)	Adjusted HR* (95% CI)	Unadjusted HR (95% CI)	Adjusted HR* (95% CI)
**Overall**						
Never smoker	1.00 (ref)	1.00 (ref)	1.00 (ref)	1.00 (ref)	1.00 (ref)	1.00 (ref)
Former smoker	0.68 (0.54–0.86)	0.86 (0.62–1.20)	0.87 (0.73–1.04)	1.19 (0.94–1.52)	1.07 (0.82–1.41)	1.21 (0.87–1.68)
Current smoker	0.50 (0.43–0.59)	0.84 (0.64–1.10)	0.50 (0.44–0.57)	1.00 (0.80–1.24)	1.01 (0.84–1.23)	1.39 (1.06–1.81)
**P interaction between race and smoking status**	0.388	0.474	0.152	0.386	< 0.001	< 0.001
**Chinese**						
Never smoker	1.00 (ref)	1.00 (ref)	1.00 (ref)	1.00 (ref)	1.00 (ref)	1.00 (ref)
Former smoker	0.79 (0.60–1.04)	1.01 (0.68–1.50)	0.95 (0.76–1.18)	1.23 (0.92–1.64)	1.16 (0.80–1.69)	1.22 (0.78–1.90)
Current smoker	0.54 (0.44–0.67)	0.90 (0.64–1.25)	0.56 (0.47–0.66)	1.09 (0.84–1.42)	1.16 (0.89–1.52)	1.57 (1.09–2.27)
**Malay**						
Never smoker	1.00 (ref)	1.00 (ref)	1.00 (ref)	1.00 (ref)	1.00 (ref)	1.00 (ref)
Former smoker	0.40 (0.23–0.71)	0.46 (0.20–1.06)	0.59 (0.40–0.86)	1.08 (0.62–1.87)	0.95 (0.56–1.62)	1.23 (0.63–2.40)
Current smoker	0.41 (0.29–0.59)	0.55 (0.29–1.05)	0.35 (0.26–0.47)	0.74 (0.44–1.24)	0.72 (0.48–1.08)	1.02 (0.61–1.71)
**Indian**						
Never smoker	1.00 (ref)	1.00 (ref)	1.00 (ref)	1.00 (ref)	1.00 (ref)	1.00 (ref)
Former smoker	0.76 (0.40–1.42)	1.12 (0.38–3.28)	0.95 (0.58–1.56)	1.40 (0.67–2.95)	1.01 (0.56–1.84)	1.13 (0.51–2.49)
Current smoker	0.44 (0.28–0.69)	1.40 (0.61–3.21)	0.47 (0.32–0.69)	1.17 (0.61–2.22)	0.95 (0.64–1.40)	1.37 (0.75–2.49)

*Adjusted for race, age, gender, history of diabetes, history of hypertension, history of hyperlipidemia, history of AMI/CABG/PCI, body mass index, Killip class on admission, CPR in ambulance/ED, anterior infarct, serum creatinine on admission, haemoglobin on admission, symptom-to-balloon time, inpatient left ventricular ejection fraction.

**AMI acute myocardial infarction, *CABG* coronary artery bypass surgery, *CPR* cardiopulmonary resuscitation, *ED* emergency department, *MI* myocardial infarction, *PCI* percutaneous coronary intervention, *STEMI* ST-elevation myocardial infarction.

Similar findings were observed in the NSTEMI group, with unadjusted HR for death significantly lower for current smokers at 30 days (HR 0.32) and at 1 year (HR 0.45) (Table 4). After adjusting for potential confounders, the protective effect of smoking disappeared. Similar 30-day and 1-year mortality results were observed when we stratified the analysis by ethnicity. Notably, the unadjusted HRs for recurrent MI within 1 year were mixed, being higher for former smokers (HR 1.39), but lower for current smokers (HR 0.68), when compared to never smokers. After adjusting for potential confounders, the adjusted HRs remained significantly higher for both former smokers (HR 1.45) and current smokers (HR 1.46), especially among Chinese patients, suggesting that current smokers have increased risk of recurrent MI following NSTEMI.

**Table 4:** Risk of death within 30 days, death within 1 year and recurrent MI within 1 year among former and current smokers compared to never smokers in NSTEMI patients.

	Death within 30 days		Death within 1 year		Recurrent MI within 1 year	
	Unadjusted HR (95% CI)	Adjusted HR* (95% CI)	Unadjusted HR (95% CI)	Adjusted HR* (95% CI)	Unadjusted HR (95% CI)	Adjusted HR* (95% CI)
**Overall**						
Never smoker	1.00 (ref)	1.00 (ref)	1.00 (ref)	1.00 (ref)	1.00 (ref)	1.00 (ref)
Former smoker	0.97 (0.70–1.35)	0.74 (0.46–1.18)	1.06 (0.87–1.30)	0.84 (0.64–1.10)	1.39 (1.16–1.66)	1.45 (1.13–1.85)
Current smoker	0.32 (0.22–0.48)	0.78 (0.45–1.35)	0.45 (0.37–0.56)	1.00 (0.74–1.35)	0.68 (0.56–0.81)	1.46 (1.13–1.89)
**P interaction between race and smoking status**	0.379	0.618	0.350	0.333	0.114	0.243
**Chinese**						
Never smoker	1.00 (ref)	1.00 (ref)	1.00 (ref)	1.00 (ref)	1.00 (ref)	1.00 (ref)
Former smoker	1.14 (0.75–1.74)	0.89 (0.49–1.63)	1.23 (0.96–1.57)	1.02 (0.74–1.42)	1.66 (1.32–2.08)	1.55 (1.14–2.11)
Current smoker	0.34 (0.20–0.58)	0.72 (0.33–1.59)	0.46 (0.34–0.61)	0.94 (0.64–1.38)	0.65 (0.50–0.84)	1.22 (0.86–1.74)
**Malay**						
Never smoker	1.00 (ref)	1.00 (ref)	1.00 (ref)	1.00 (ref)	1.00 (ref)	1.00 (ref)
Former smoker	0.45 (0.19–1.03)	0.30 (0.09–0.94)	0.71 (0.44–1.14)	0.50 (0.25–1.01)	1.00 (0.66–1.51)	1.37 (0.75–2.48)
Current smoker	0.18 (0.07–0.43)	0.57 (0.17–1.93)	0.33 (0.21–0.53)	0.85 (0.40–1.80)	0.54 (0.36–0.80)	1.68 (0.87–3.23)
**Indian**						
Never smoker	1.00 (ref)	1.00 (ref)	1.00 (ref)	1.00 (ref)	1.00 (ref)	1.00 (ref)
Former smoker	1.19 (0.58–2.46)	0.90 (0.29–2.76)	0.93 (0.54–1.57)	0.48 (0.22–1.05)	1.14 (0.76–1.72)	1.23 (0.68–2.22)
Current smoker	0.41 (0.18–0.93)	0.87 (0.26–2.95)	0.55 (0.34–0.90)	1.08 (0.53–2.20)	0.80 (0.56–1.15)	1.64 (0.97–2.77)

*Adjusted for race, age, gender, history of diabetes, history of hypertension, history of hyperlipidemia, history of AMI/CABG/PCI, body mass index, Killip class on admission, CPR in ambulance/ED, serum creatinine on admission, haemoglobin on admission, inpatient left ventricular ejection fraction.

**AMI acute myocardial infarction, *CABG* coronary artery bypass surgery, *CPR* cardiopulmonary resuscitation, *ED* emergency department, *MI* myocardial infarction, *NSTEMI* non ST-elevation myocardial infarction, *PCI* percutaneous coronary intervention.

## Discussion

In this national registry-based study of a multi-ethnic cohort of AMI patients treated by PCI, we found that former and current smokers had a decreased unadjusted HR for both 30-day and 1-year mortality, when compared to never smokers. However, this protective effect of smoking was not present after adjustment of potential confounding factors, suggesting the existence of a smokers’ pseudoparadox on mortality for both STEMI and NSTEMI. We found an increased adjusted HR for recurrent MI within 1 year among current smokers in the STEMI group and the Chinese STEMI sub-group. Similarly, we found that the adjusted risk for recurrent MI within 1 year were increased among both former and current smokers in the NSTEMI group.

The smoker’s paradox was first described in 1968 by Weinblatt et al. as an unexpected result because the smokers had a lower 1 month mortality after their MI events as compared to non-smokers in the report^20^. This paradoxical result was observed in another independent study by Tamsin Lisa et al. where the smokers had a lower prevalence for AMI and other CVDs, such as hypertension, congestive heart failure and angina pectoris when logistic regression was performed^21^. In this paper, the authors noted that smokers were about 10 years younger than the non-smokers and this paradoxical result was presumably due to the age difference, given that age is one of the most important factors for developing CVDs and predisposes to AMI events via generation of reactive oxygen species (ROS), cellular senescence, and epigenetic changes^22–24^.

Many of the former studies which attempted to elucidate the association of smoking with clinical outcomes of AMI had intrinsic limitations. For example, one of the earlier studies using data from three hospitals reported the protective effect of smoking on the outcomes of acute STEMI^8^. However, this study did not have age adjustment despite the significant difference in age between smokers and non-smokers which makes data interpretation difficult. Similarly, in another study where national Malay data were utilized, the study reported a positive correlation between smoking and better outcomes for both STEMI and NSTEMI patients^7^. This paper was one of the few studies which utilized the national dataset and reported both STEMI and NSTEMI separately. However, this study again did not include adjustment for age even though there was about 9 years of difference in age between smokers and non-smokers, suggesting that the lack of age adjustment could possibly impact the data interpretation. Therefore, the abovementioned epidemiological studies which demonstrated the protective association of smoking with the outcomes of AMI may be confounded by the lack of statistical adjustment for age.

Two recent papers have reported the detrimental effect of smoking on the AMI outcomes. In the national dataset of Chinese cohort, Gao et al*.* found that without the proper adjustments for age and the number of cigarettes smoked, the smoking history was associated with a better outcome in the MI patients^25^. However, when the number of cigarettes smoked and age are considered, the same dataset showed a significantly negative correlation of smoking with clinical outcomes in the MI patients. Similarly, independent groups reported that smoking was associated with an increased risk of all-cause mortality and heart failure from meta-analysis of PCI trials^26,27^. However, these two studies had some limitations as the former study did not subclassify AMI patients into STEMI and NSTEMI and was conducted only in patients with Chinese ethnicity. Similarly, the latter study analyzed STEMI patients who participated in clinical trials, suggesting that the patient population might not be generalizable. Our present report contributes further to the field by elucidating the detrimental effect of smoking on clinical outcomes of multi-ethnic STEMI and NSTEMI patients as our study utilized data of a national registry with appropriate statistical adjustment for potential confounders.

In our dataset, it was consistently observed that both STEMI and NSTEMI smokers were at higher risk of recurrent MI within 1 year. This result is consistent with the former reports by Redfors and Gao et al.as they also reported an increased risk of recurrent MI by smoking^25,27^. Interestingly, despite the increase in recurrent MI, an increased all-cause mortality was not observed in our data analysis. This was an unexpected result as other groups observed both increased recurrent MI and all-cause mortality^25^ and the recurrent MI is one of the strongest predictors for an increased all-cause morality^28,29^. This phenomenon could be due to other factors, such as advances in the health care system or the short travel time between the patients’ houses and the hospitals in Singapore due to urbanization which would lead to better mortality^30–34^. Another interesting result we found was that the former Malay smokers had a better clinical outcome in terms of death within 30 days after NSTEMI events with a hazard ratio of 0.30. This result may indicate that for this specific group, there were confounding factors other than age.

The detrimental association between smoking status and the clinical outcomes was observed in the overall and Chinese group for both STEMI and NSTEMI, but not in the Malay and Indian groups (Tables 3 and 4). This result suggests that the differential effect of smoking on the prognosis of STEMI and NSTEMI may be influenced by ethnicity. Similarly, other groups previously reported that the risk of smoking on AMI events was dependent on the types of small nucleotide polymorphism (SNP) on certain genes, such as Paraoxonase (PON1) and rs1122608^35,36^. In other words, these SNPs may determine whether smoking increases the susceptibility of subjects to AMI. The SNPs that are be associated with the worse prognosis by smoking in our cohort and the mechanisms by which smoking interacts with these SNPs need to be experimentally identified and validated in the future.

There is indirect evidence suggesting the detrimental role of smoking on AMI. For example, cases of AMI significantly decreased after the introduction of national smoking bans in multiple countries, suggesting that smoking increases the risk of AMI^37–39^. Moreover, the percentage of smokers among AMI patients was significantly greater as compared to non-AMI patients^40^. Considering the fact that cigarette smoking is attributable to 15% of the CVD cases and mortality^41^ and increases the risk of MI, smoking should be strictly avoided. Additionally, there are still unanswered questions on whether second-hand smoking or cigarette alternatives like e-cigarettes also lead to poorer outcomes in MI patients.

A limitation of the study is that the SMIR database does not contain information on the duration of smoking and the number of cigarettes the smokers consume. Additionally, the SMIR does not include the information on how long it has been after the former smokers quit their smoking behavior. For that reason, patients who identified as former smokers were classified as such unlike the WHO definition which requires 12 months of cessation to qualify one as a former smoker^42^. Hence, the results from our study may not be directly comparable with other studies due to the different definition. Furthermore, due to the lack of information on the number of cigarettes smoked, we could not examine for the presence of any potential dose–response relationship between smoking and outcomes.

## Conclusion

In summary, we found that smokers seemingly had better clinical outcomes (30-day and 1-year mortalities) after STEMI or NSTEMI. However, upon adjustment, the seemingly beneficial effects of smoking on mortality disappeared and the risk of recurrent MI within 1-year was significant higher in STEMI and NSTEMI smokers, confirming the presence of a smokers’ pseudoparadox for mortality. This data demonstrates that the previously reported protective effect of smoking was actually pseudo-protective and smoking worsens clinical outcomes in both STEMI and NSTEMI patients.

## Supplementary Information

Supplementary Information

## References

[1] Dagenais GR (2020). Variations in common diseases, hospital admissions, and deaths in middle-aged adults in 21 countries from five continents (PURE): A prospective cohort study. Lancet.

[2] Fuster, V. & Kelly, B. B. *Promoting cardiovascular health in the developing world: A critical challenge to achieve global health*. *Promoting Cardiovascular Health in the Developing World: A Critical Challenge to Achieve Global Health* (National Academies Press, 2010). doi:10.17226/12815 20945571

[3] Bhatnagar P, Wickramasinghe K, Wilkins E, Townsend N (2016). Trends in the epidemiology of cardiovascular disease in the UK. Heart.

[4] Roth GA (2015). Demographic and epidemiologic drivers of global cardiovascular mortality. N. Engl. J. Med..

[5] Puymirat E (2017). Acute myocardial infarction: Changes in patient characteristics, management, and 6-month outcomes over a period of 20 years in the FAST-MI program (French registry of acute ST-elevation or non-ST-elevation myocardial infarction) 1995 to 2015. Circulation.

[6] Oliveira, A., Barros, H., Júlia Maciel, M. & Lopes, C. Tobacco smoking and acute myocardial infarction in young adults: A population-based case-control study. *Prev. Med. (Baltim).* **44**, 311–316 (2007). 10.1016/j.ypmed.2006.12.002 17239433

[7] Venkatason, P. *et al.* The bizzare phenomenon of smokers’ paradox in the immediate outcome post acute myocardial infarction: an insight into the Malaysian National Cardiovascular Database-Acute Coronary Syndrome (NCVD-ACS) registry year 2006–2013. *Springerplus* **5**, (2016). 10.1186/s40064-016-2188-3 PMC4846599 27186498

[8] Symons R (2016). Impact of active smoking on myocardial infarction severity in reperfused ST-segment elevation myocardial infarction patients: The smoker’s paradox revisited. Eur. Heart J..

[9] Wittmann F (2018). To be or not to be: the “smoker’s paradox”: An in-vitro study. Cell. Physiol. Biochem..

[10] Gagne, J. J. *et al.* Effect of smoking on comparative efficacy of antiplatelet agents: Systematic review, meta-analysis, and indirect comparison. *BMJ* **347**, (2013). 10.1136/bmj.f5307 PMC3775704 24046285

[11] Nakada Y (2017). Hypoxia induces heart regeneration in adult mice. Nature.

[12] Stone GW (2016). Relationship between infarct size and outcomes following primary PCI patient-level analysis from 10 randomized trials. J. Am. Coll. Cardiol..

[13] Yang X, Cohen MV, Downey JM (2010). Mechanism of cardioprotection by early ischemic preconditioning. Cardiovasc. Drugs Ther..

[14] Yellon DM, Downey JM (2003). Preconditioning the myocardium: From cellular physiology to clinical cardiology. Physiol. Rev..

[15] McMechan SR, Adgey AAJ (1998). Age related outcome in acute myocardial infarction. BMJ.

[16] Yeo, Y. *et al. Singapore Myocardial Infarction Registry Annual Report 2018 National Registry of Diseases Office Acknowledgement*. (2020).

[17] Ho AFW (2016). Emergency medical services utilization among patients with ST-segment elevation myocardial infarction: Observations from the Singapore myocardial infarction registry. Prehospital Emerg. Care.

[18] Lu, H. T. & Nordin, R. B. Ethnic differences in the occurrence of acute coronary syndrome: Results of the Malaysian National Cardiovascular Disease (NCVD) Database Registry (March 2006 - February 2010). *BMC Cardiovasc. Disord.* **13**, (2013). 10.1186/1471-2261-13-97 PMC4229312 24195639

[19] Fine JP, Gray RJ (1999). A proportional hazards model for the subdistribution of a competing risk. J. Am. Stat. Assoc..

[20] Weinblatt E, Shapiro S, Frank CW, Sager RV (1968). Prognosis of men after first myocardial infarction: Mortality and first recurrence in relation to selected parameters. Am. J. Public Health Nations. Health.

[21] Tamsin Lisa, K., Gilpin, E., Ahnve, S., Henning, H. & Ross, J. Smoking status at the time of acute myocardial infarction and subsequent prognosis. *Am. Heart J.* **110**, 535–541 (1985). 10.1016/0002-8703(85)90071-7 4036780

[22] Tanik VO (2019). The predictive value of PRECISE-DAPT score for in-hospital mortality in patients with ST-elevation myocardial infarction undergoing primary percutaneous coronary intervention. Angiology.

[23] Çinar T (2019). The predictive value of age, creatinine, ejection fraction score for in-hospital mortality in patients with cardiogenic shock. Coron. Artery Dis..

[24] Hubbard R (2005). Use of nicotine replacement therapy and the risk of acute myocardial infarction, stroke, and death. Tob. Control.

[25] Gao K, Shi X, Wang W (2017). The life-course impact of smoking on hypertension, myocardial infarction and respiratory diseases. Sci. Rep..

[26] Yadav M (2019). The Smoker’s Paradox Revisited: A Patient-Level Pooled Analysis of 18 Randomized Controlled Trials. JACC Cardiovasc. Interv..

[27] Redfors B (2020). Effect of smoking on outcomes of primary PCI in patients with STEMI. J. Am. Coll. Cardiol..

[28] Cao CF, Li SF, Chen H, Song JX (2016). Predictors and in-hospital prognosis of recurrent acute myocardial infarction. J. Geriatr. Cardiol..

[29] Thune JJ (2011). Predictors and prognostic impact of recurrent myocardial infarction in patients with left ventricular dysfunction, heart failure, or both following a first myocardial infarction. Eur. J. Heart Fail..

[30] León-Jiménez, C. *et al.* Hospital arrival time and functional outcome after acute ischaemic stroke: Results from the PREMIER study. *Neurol. (English Ed.* **29**, 200–209 (2014). 10.1016/j.nrl.2013.05.003 24021783

[31] Wilkinson J (2002). Interaction between arrival time and thrombolytic treatment in determining early outcome of acute myocardial infarction. Heart.

[32] Guan, W. *et al.* Time to hospital arrival among patients with acute myocardial infarction in China: A report from China PEACE prospective study. *Eur. Hear. J. Qual. Care Clin. Outcomes* **5**, 63–71 (2019). 10.1093/ehjqcco/qcy022 PMC6307335 29878087

[33] Shiraishi Y (2015). Time interval from symptom onset to hospital care in patients with acute heart failure: A report from the Tokyo cardiac care unit network emergency medical service database. PLoS ONE.

[34] Gamble J-M (2011). Patterns of care and outcomes differ for urban versus rural patients with newly diagnosed heart failure, even in a universal healthcare system. Circ. Hear. Fail..

[35] Chen QF (2018). Correlation of rs1122608 SNP with acute myocardial infarction susceptibility and clinical characteristics in a Chinese Han population: A case-control study. Anatol. J. Cardiol..

[36] Senti M, Aubo C, Tomas M (2000). Differential effects of smoking on myocardial infarction risk according to the Gln/Arg 192 variants of the human paraoxonase gene. Metabolism.

[37] Lin H (2013). The effects of smoke-free legislation on acute myocardial infarction: A systematic review and meta-analysis. BMC Public Health.

[38] Gao M (2019). The effect of smoke-free legislation on the mortality rate of acute myocardial infarction: A meta-analysis. BMC Public Health.

[39] Carrión-Valero F (2020). Association between a comprehensive smoking ban and hospitalization for acute myocardial infarction: An observational study in the Autonomous Community of Valencia Spain. Rev. Port. Cardiol..

[40] Jing M (2016). Comparison of long-term mortality of patients aged ≤ 40 versus > 40 years with acute myocardial infarction. Am. J. Cardiol..

[41] Yusuf S (2020). Modifiable risk factors, cardiovascular disease, and mortality in 155 722 individuals from 21 high-income, middle-income, and low-income countries (PURE): A prospective cohort study. Lancet.

[42] World Health Organization (2009). Global Adult Tobacco Survey.

