# Supplementary material for: Association between smoking status and outcomes in myocardial infarction patients undergoing percutaneous coronary intervention
Source: Sci Rep. 2021 Mar 19;11:6466. doi: 10.1038/s41598-021-86003-w (PMC7979717; doi:10.1038/s41598-021-86003-w)
Supplement: Supplementary file 1 — Supplementary Information [file 41598_2021_86003_MOESM1_ESM.pptx]

## Slide 1
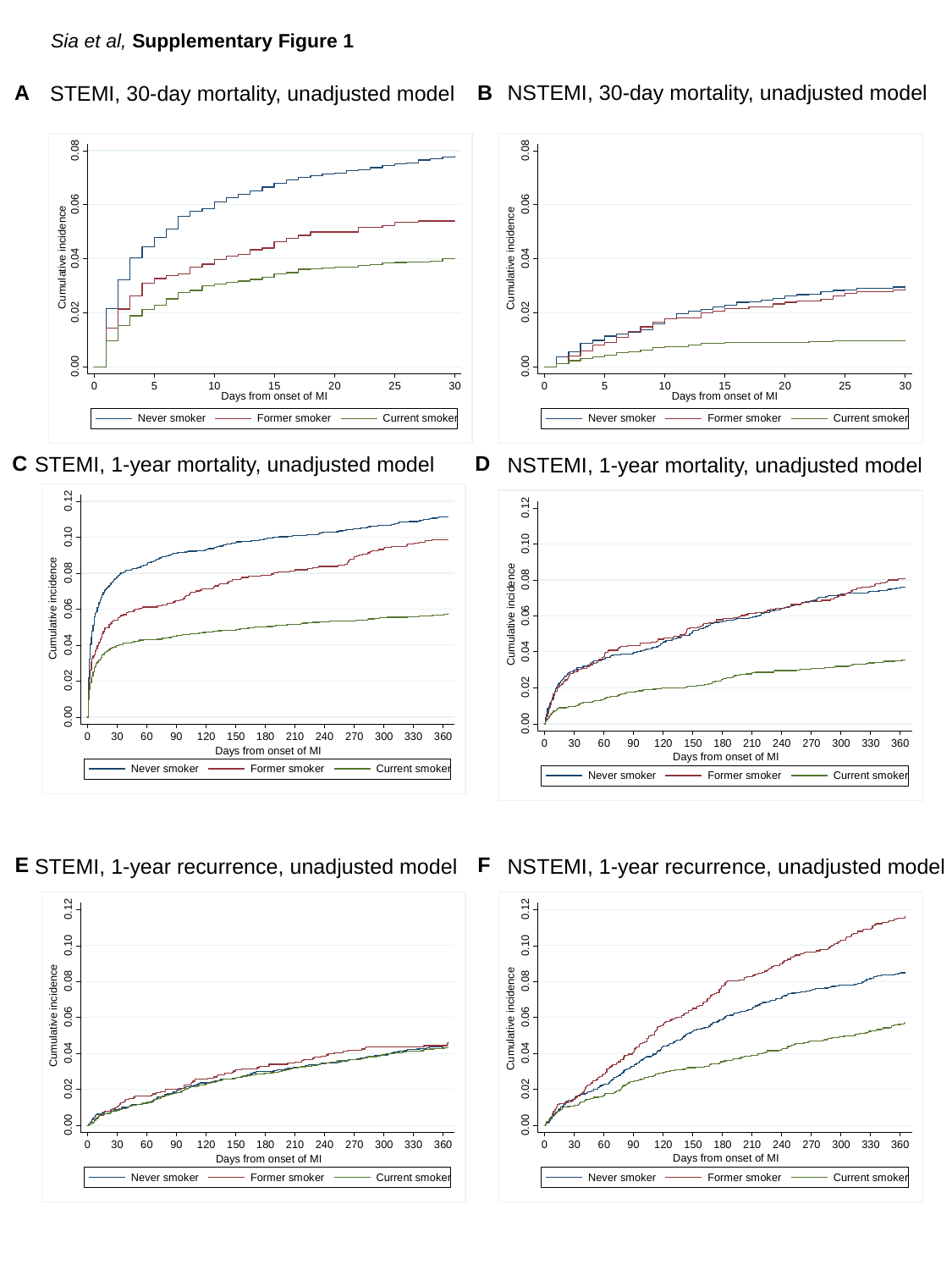

Sia et al, Supplementary Figure 1
NSTEMI, 30-day mortality, unadjusted model
STEMI, 30-day mortality, unadjusted model
A
B
STEMI, 1-year mortality, unadjusted model
NSTEMI, 1-year mortality, unadjusted model
C
D
STEMI, 1-year recurrence, unadjusted model
NSTEMI, 1-year recurrence, unadjusted model
E
F
